# Assessing the Efficacy of Tragal Pumping in a Novel Tympanostomy Tube‐Rat Model

**DOI:** 10.1002/oto2.160

**Published:** 2024-07-06

**Authors:** Satoshi Hara, Xinyu Liu, Firasat Ali Shah, Harumi Saeki, Hajime Orita, Airi Sakyo, Takashi Anzai, Yusuke Takata, Kazusaku Kamiya, Fumihiko Matsumoto, Kathleen Gabrielson

**Affiliations:** ^1^ Department of Molecular and Comparative Pathobiology Johns Hopkins University School of Medicine Baltimore Maryland USA; ^2^ Department of Otorhinolaryngology Juntendo University Faculty of Medicine Tokyo Japan; ^3^ International Collaborative Research Administration Juntendo University Tokyo Japan; ^4^ Department of Human Pathology Juntendo University Faculty of Medicine Tokyo Japan; ^5^ Department of Gastroenterology and Minimally Invasive Surgery Juntendo University Faculty of Medicine Tokyo Japan; ^6^ Department of Otorhinolaryngology, Graduate School of Medicine Juntendo University Tokyo Japan

**Keywords:** eardrop, intratympanic injection, middle ear, tragal pumping, tympanostomy tube

## Abstract

**Objective:**

Tragal pumping (TP) is a practice of pushing on the tragus to raise pressure within the external auditory canal and is a commonly recommended adjunctive maneuver believed to facilitate the introduction of ototopical medications into the middle ear cavity via a tympanostomy tube. To investigate the efficacy of TP in the penetration of eardrops into the middle ear cavity via tympanostomy tube, we established the novel tympanostomy tube‐rat model. We investigated the histology of the middle ear to determine the efficacy in moving fluid into the middle ear.

**Study Design:**

Prospective controlled animal study.

**Setting:**

Animal laboratory in a university hospital.

**Methods:**

Ten rats were recruited, and a tympanostomy tube insertion and green dye eardrops into outer ears were performed on bilateral ears. TP was performed only on 1 ear and was not applied on the other ear in each rat. Green dye in a middle ear cavity in hematoxylin and eosin‐stained temporal bone sections was evaluated by blinded experts in microscopic anatomy (staining grade) and by using Image J software (staining level). The results of these 2 methods were statistically validated.

**Results:**

The staining grade (*P* < .001) and the staining level (*P* < .001) were significantly higher in the ears which we applied TP than in the control ears. The results of 2 methods were significantly and positively correlated (*r* = .898, *P* < .001).

**Conclusion:**

Our results showed that the TP accelerate the penetration of eardrops into the middle ear cavity in the tympanostomy tube‐rat model.

Tragal pumping (TP) is a practice of pushing on the tragus to raise the pressure within the external auditory canal (EAC) and is a commonly recommended adjunctive maneuver believed to facilitate the introduction of ototopical medications into the middle ear cavity via a tympanostomy tube.^1^ It is reported that TP can create enough pressure between EAC and the middle ear cavity to propel most liquids through a tympanostomy tube.^2^

In comparison, another method used to introduce drugs into the middle ear is through intratympanic injection (ITI). This technique is also frequently used by otolaryngologists to inject a drug into middle ear cavity to treat middle and inner ear diseases such as idiopathic sudden sensorineural hearing loss (ISSNHL),^3^ Menière's disease (MD),^4^ and otitis media with effusion.^5^ Animal studies have demonstrated that the drug injected into the middle ear cavity resulted in significantly higher perilymph drug concentrations than intravenous or oral administration.^6^, ^7^, ^8^, ^9^ ITI with steroid or gentamicin is presented as a recommendation by the American Academy of Otolaryngology–Head and Neck Surgery guideline for ISSNHL or MD, respectively.^3^, ^4^ On the other hand, the guidelines mention the protocols of ITI therapy as up to 4 sessions within 2 weeks.^3^, ^4^ Therefore, ITI potentially results in a substantial burden on patients and health care services because patients need to visit a clinic and undergo ITI multiple times in a short time frame, therefore, an alternative method of ITI has been desired.

TP with tympanostomy tube insertion and eardrops may be a potential alternative method of ITI for the ototopical drug delivery into the middle ear cavity to reduce the burden on patients and health care services based on previous human studies.^10^, ^11^ It is reported that solutions do move to the middle ear with the use of the TP through the tympanostomy tube but without TP, solutions do not move well to the middle ear in a randomized human study and an ex vivo human ear model.^1^, ^12^ However, the information of the efficacy and histology of the middle ear and temporal bone after TP has not been described in vivo in any model.

Figure 1 shows the tragus in a rat. To elucidate the efficacy of the TP in a rat, we established a novel rat model with the tympanostomy tube (internal diameter [ID]: 1.14 mm) which is the same size as the one used in human studies^10^, ^11^, and we investigated the histology of the middle ear/temporal bone preparation in the rat model. There are some reports about a tympanostomy tube‐rat model using small tympanostomy tubes (ID: 0.28‐0.76 mm),^13^, ^14^, ^15^ however, a larger tube was used in the current study in an adult rat model. Because the ID of the tube can affect the penetration of solutions via the tympanostomy tube,^16^ the tube which is used in humans would be more instructive to investigate. Hence, we investigated the efficacy of TP with the larger tube in the rat model.

**Figure 1:**
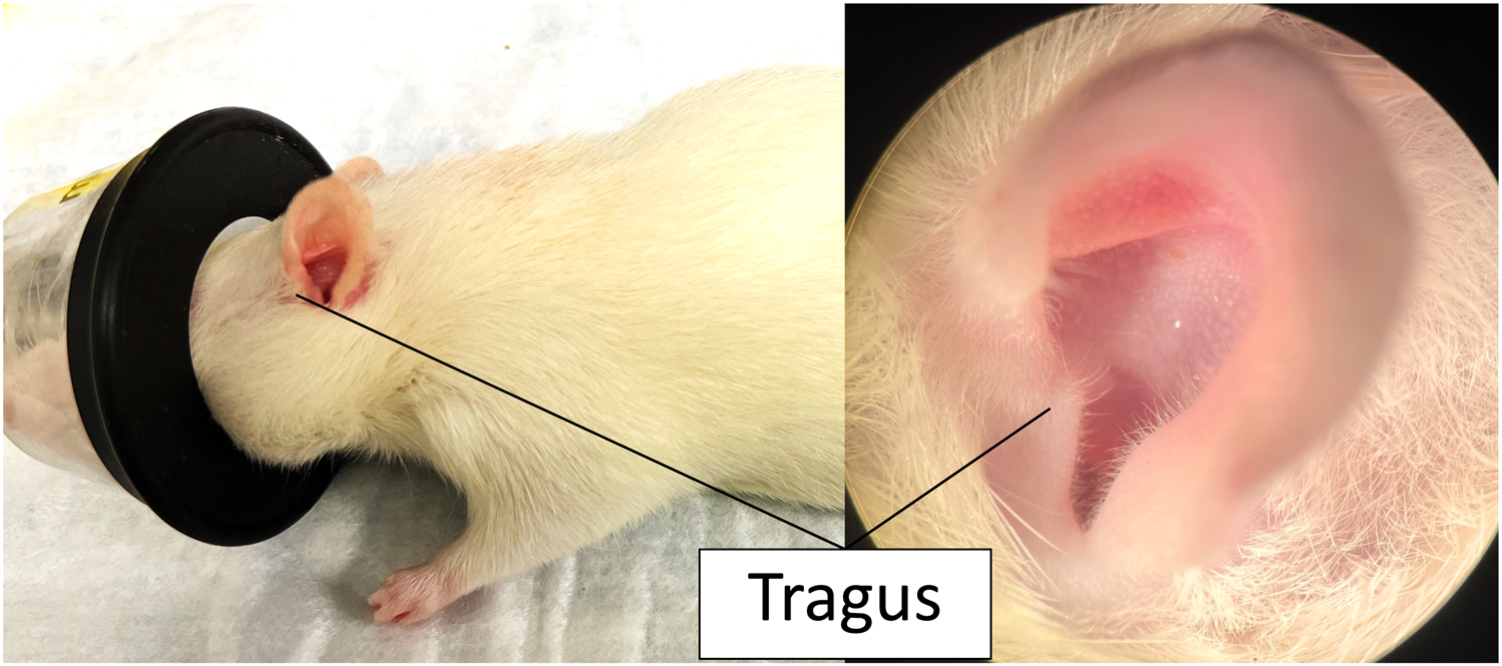
Anatomy of tragus in a rat.

## Materials and Methods

All the procedures and experiments involving rats were performed according to protocols approved by the Johns Hopkins University Animal Care and Use Committee (Approval Number: RA23M358).

### Experimental Design

For all rats, tympanostomy tube insertion and dye ear drops into outer ears were performed on bilateral ears while rats were under isoflurane general anesthesia. Thereafter, to minimize confounding, to investigate the efficacy of TP, TP was performed only on 1 ear (TP ear) in a rat and was not applied on the other ear (control ear) in the same rat. The ear for the TP ear was randomly chosen in each rat.

### Animals

Sprague‐Dawley rats were housed in standard polycarbonate rat cages in a temperature and humidity‐controlled room under a 14:10 hours light/dark cycle, lights on 07:00 to 21:00 hours. The animals were allowed access to water and standard chow ad libitum. The ages of 6 to 8 months male and female adult breeder rats (n = 10) were assigned to this study.

### Anesthesia

Rats were placed in a medium‐sized induction chamber for induction of anesthesia with 2% to 3% isoflurane. Animals were monitored for the depth of anesthesia by reflex‐to‐toe pinch and respiratory rate. Rats were maintained with anesthesia via a nosecone with ∼1.5% to 2% isoflurane. Eye lubricant was placed in both the eyes of the rat to prevent corneal desiccation caused by anesthesia. The rat was placed on a low heat setting heating pad to ensure a normal body temperature (37°C).

### Tympanostomy Tube Insertion

**Figure 2:**
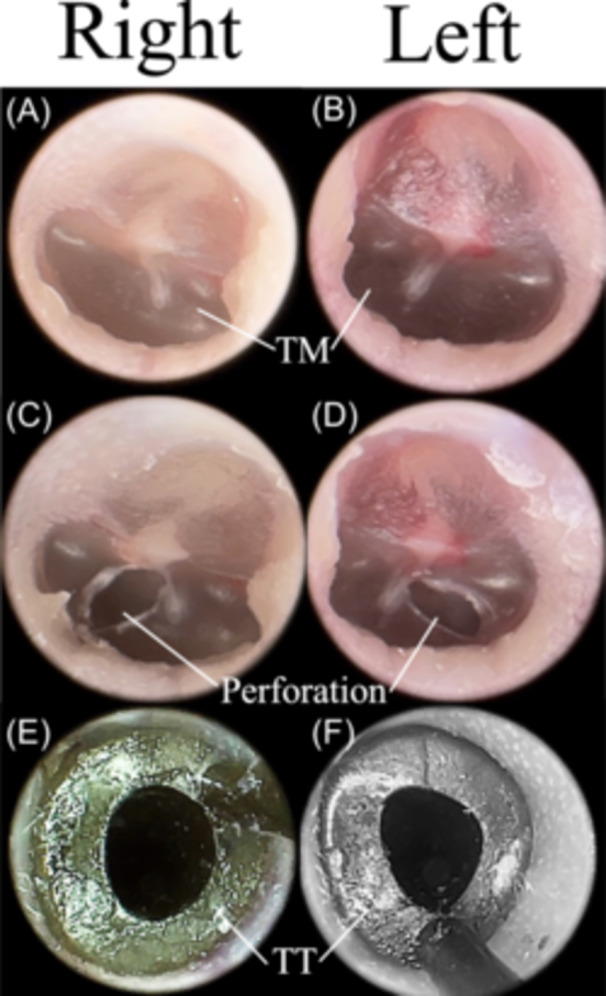
Representative endoscopic photos of the rat ears. (A) Right tympanic membrane before the procedures. (B) Left tympanic membrane before the procedures. (C) Right tympanic membrane with a perforation after myringotomy. (D) Left tympanic membrane with a perforation after myringotomy. (E) Right ear after tympanostomy tube insertion. (F) Left ear after tympanostomy tube insertion. TM, tympanic membrane; TT, tympanostomy tube.

Using a microscope and a 2.7 mm diameter 0° endoscope (MEDIT Inc), both ears were examined to exclude outer and middle ear diseases (Figure 2A and B). Earwax from the EAC was removed when needed. Small incisions were placed with Beaver Spear Tip Myringotomy Knife (Beaver‐Visitec International) on the posterior half of pars tensa of bilateral tympanic membranes, which is described in previous studies.^13^, ^15^ (Figure 2C and D) Bilateral tympanostomy tubes (Silicone Shepard Ventilation Tube, 1026025, ID: 1.14 mm; Medtronic), which are the same size as the one used in previous human studies,^10^, ^11^ were inserted into the tympanic membrane perforations with micro alligator ear forceps (Nagashima Medical Instruments Co). The placement of the tympanostomy tubes was confirmed with the endoscope (Figure 2E and F).

### Confirmation of the Placement of Tympanostomy Tube Insertion

**Figure 3:**
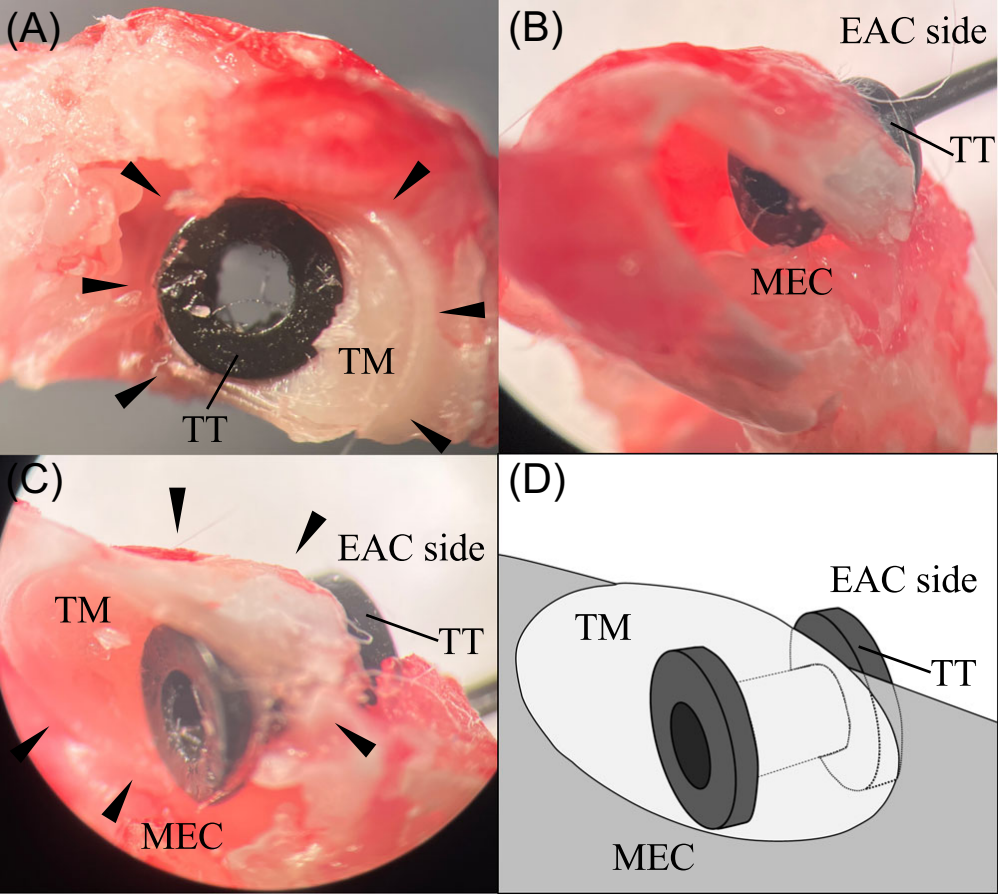
Microscopic images and an illustration of a temporal bone for the confirmation of the placement of tympanostomy tube insertion. (A) Lateral view of the temporal bone from the EAC side. The tympanostomy tube was on the tympanic membrane. (B, C) After opening the middle ear cavity, the tympanostomy tube passed through the tympanic membrane. One flange of the tube was in EAC, and the other one was in MEC. (D) An illustration of (C). EAC, external auditory canal; MEC, middle ear cavity; TM, tympanic membrane; TT, tympanostomy tube.

Since this tympanostomy tube (ID: 1.14 mm) placement had never been described in a rat, we first confirmed its placement and utilization because the tympanic membrane could not be seen clearly after the tympanostomy tube insertion. We did a pilot experiment after the tympanostomy tubes were inserted and the temporal bone was harvested leaving the tympanostomy tube in place. The dissecting microscopic analysis showed that the tympanostomy tubes were correctly placed in the tympanic membrane penetrating into the middle ear cavity (Figure 3).

### Ear Drops and TP for TP Ear

For the TP ear procedure, the rats were laid in the lateral position with the TP ear facing upward. To detect the penetration of eardrop via a tympanostomy tube, green Davidson® tissue marking dye (1163‐1; Bradley Products Inc) was diluted (1:2) with distilled water and used as eardrops. A hundred microliter of dye eardrop solution was applied into EAC with a 22G catheter connected to a 1 mL syringe with application done under microscopic review. Regarding the volume of the solution that we applied, although the volume of the middle ear cavity of a rat is reported as 42 to 82 µL,^16^, ^17^, ^18^ the rats in these studies are younger than those in our study. Moreover, a volume of 100 µL can be injected to the middle ear cavity of a rat in previous studies,^19^, ^20^ therefore, 100 µL of the solution was used in this study.

Thereafter, the tragus was gently pushed repeatedly for 1 minute. Thirty minutes after applying the eardrops, the eardrops in the EAC were removed with the same size of catheter and syringe, and then the tympanostomy tube was also removed with the ear forceps using the microscope.

### Ear Drops for Control Ear

For control ear, the same procedure was done except without performing TP. The rats were laid in the lateral position with the control ear upward. Thirty minutes after applying the dye eardrop solution into the EAC, the eardrop in the EAC and the tympanostomy tube were removed. Thereafter, we confirmed that there was no solution in the EAC that spilled from the middle ear cavity under the microscope. The rats were euthanized immediately after all procedures described above. The rats were under general anesthesia until euthanasia. The procedures for TP ears were performed first to prevent the control ear from losing the stain during the procedures.

### Histological Analyses

**Figure 4:**
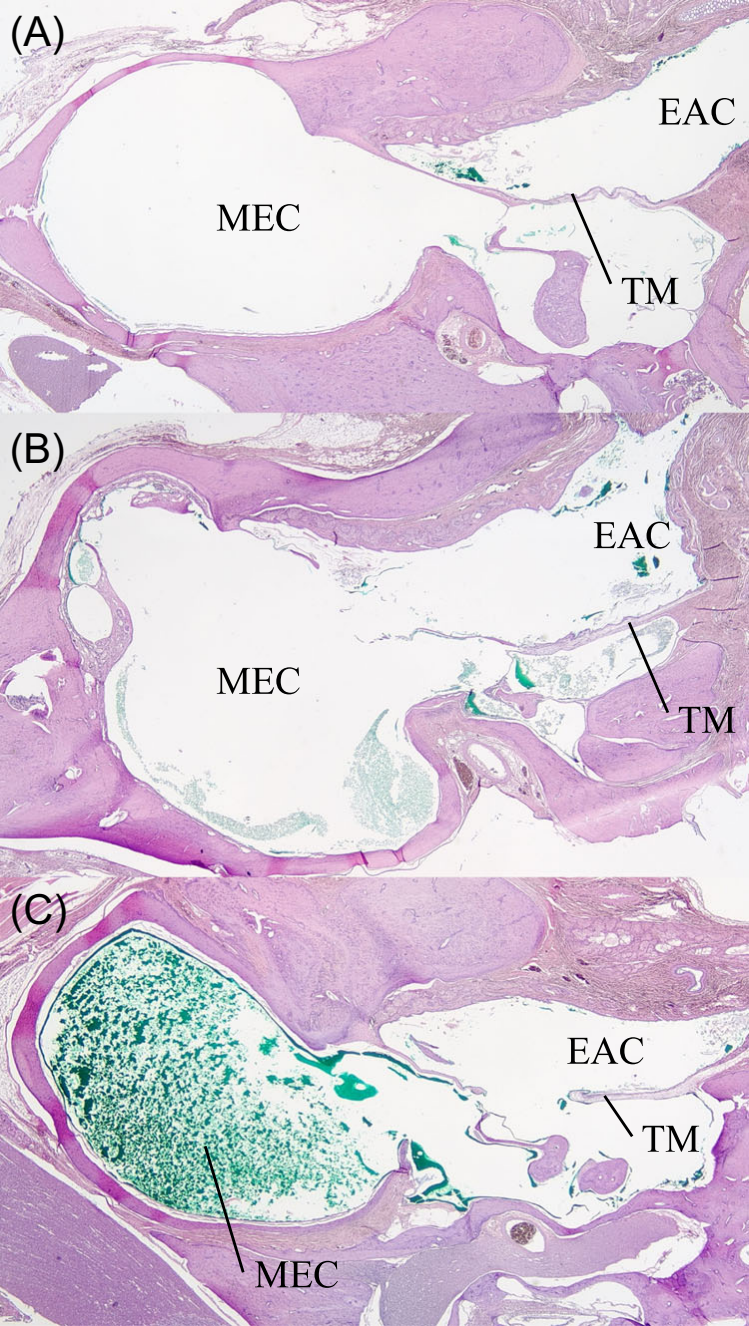
Representative pictures of temporal bone sections in each staining grade. (A), Grade 1. (B), Grade 2. (C) Grade 3. EAC, external auditory canal; MEC, middle ear cavity; TM, tympanic membrane.

After euthanasia, both temporal bones were harvested from the rats for histologic analysis. Briefly, after the skull was removed from the body, the skull around the EAC was exposed, the cartilaginous EAC was cut near the tympanic membrane, then the muscles and bones were trimmed around the temporal bone to isolate the middle ear and temporal bone. On a shaker, the temporal bone preparation was immersed for 24 hours in a volume of decalcification solution (Statlab) equal to ≥10 times the size of the tissue. After decalcification, the tissues were rinsed for 3 minutes in flowing deionized water and kept in a 10% buffered formaldehyde solution for ≥24 hours. Tissues for histology processing were placed in tissue cassettes to highlight the middle ear on a cross section. Multiple thin sections were cut horizontally from each tissue block and the 1 section per animal that showed the largest middle ear cavity was used for this analysis. Standard histology tissue processing was done, and hematoxylin and eosin staining was performed. The green stain by dye eardrop solution in the middle ear cavity was graded microscopically by a pathologist blinded to the 2 groups using a scale of 1 to 3. The grading was as follows: 1 = middle ear cavity with less than 5% stained; 2 = 5% to 40% stained; 3 = over 40% stained in the middle ear cavity. Figure 4 showed representative pictures of temporal bone sections in each grade. To reduce potential subjectivity in this analysis, animal procedure information was not included during grading.

### Image Analysis

**Figure 5:**
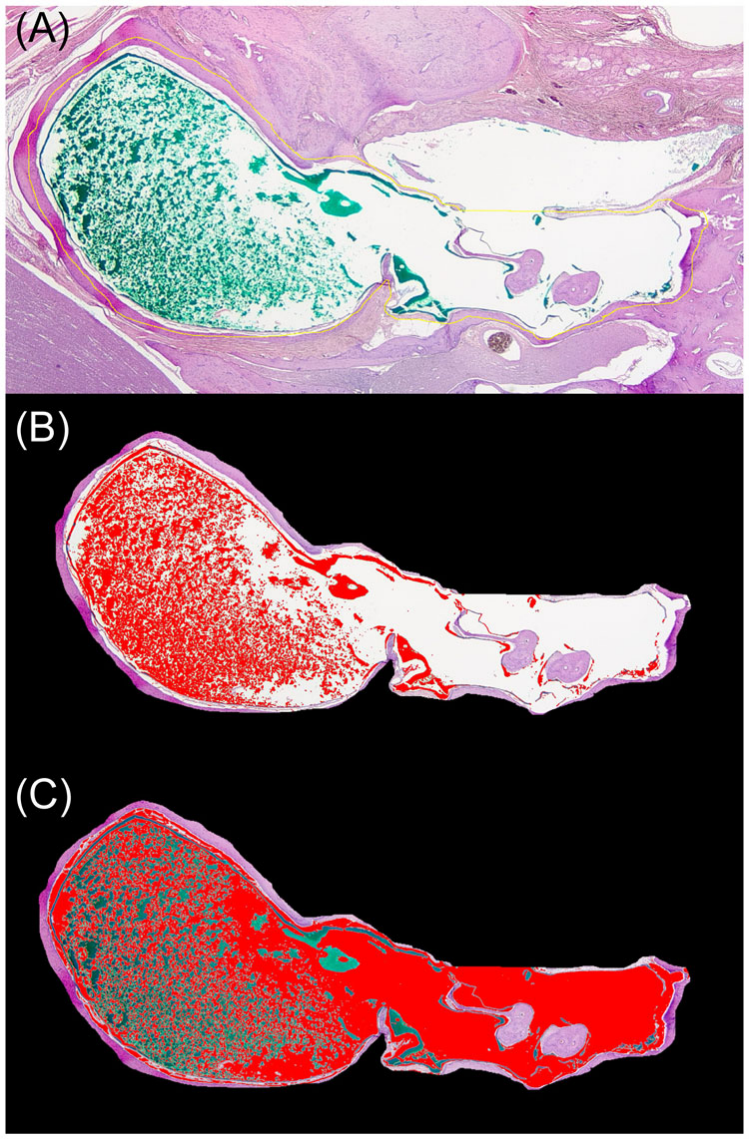
Representative images of temporal bone sections during the image analysis by a blinded scorer using Image J software. (A) Process of cropping middle ear cavity. The yellow line is a cropping line. (B) Process of selecting the green dyed area in the middle ear cavity specifically. The red area in this picture is the selected area. (C) Process of selecting the white undyed area in the middle ear cavity specifically. The red area in this picture is the selected area.

To objectively compare the efficacy of TP and the staining in the middle ear, the histology slides were analyzed using Image J software methodologies without animal procedure information knowledge. Photographs of the area of the middle ear cavity were analyzed by a blinded scorer. The dyed area and undyed area were measured with the color threshold function as follows. The thresholds used in this study were L: 1 to 220, A: 0 to 130, B: 0 to 255 for green dyed area, and L: 220 to 255, A: 0 to 130, B: 0 to 255 for white undyed area (Figure 5). The staining level (%) was calculated as (dyed area)/(dyed area + undyed area).

### Statistical Analyses

Descriptive statistics were presented as means ± standard deviations or medians (interquartile ranges) for the continuous variables. The Mann‐Whitney *U* tests were used to compare the variables. Pearson correlation analysis was used to evaluate the relationship of the variables. A *P* value < .05 was considered statistically significant. All analyses were performed with SPSS Statistics version 28 (IBM).

## Results

### Animal Profiles

Five males and 5 females were used in this study. The mean ± standard deviation of age was 235.2 ± 14.8 days and of weight was 357.2 ± 107.5 g. TP was applied to the left ear in 5 rats and was applied to the right ear in the other 5 rats.

### Histological Analysis

**Figure 6:**
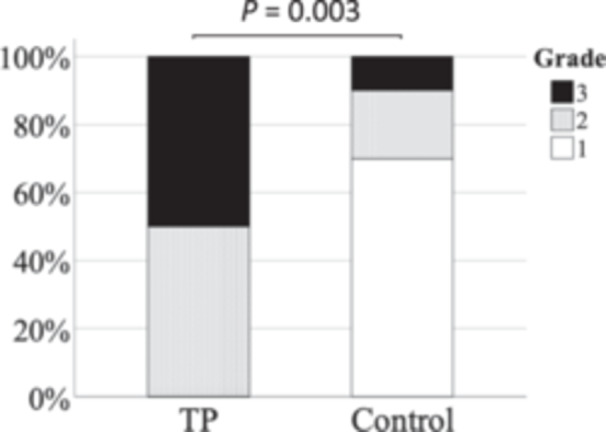
The distribution of the staining grades of the middle ear cavity in the TP and control ears. TP, tragal pumping.

Figure 6 shows the distribution of the staining grades in the TP and control ears. In the TP ears, there were (5) grade 3, (5) grade 2, and (0) grade 1. In the control ears, there were (1) grade 3, (2) grade 2, and (7) grade 1. Mann‐Whitney *U* test showed that the staining grades were significantly higher in the TP ears than in the control ears (*P* = .003).

### Image Analysis

**Figure 7:**
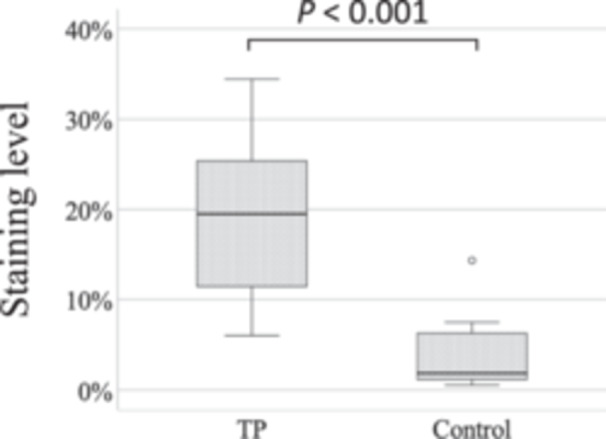
The staining level of the middle ear cavity in the TP and control ears. TP, tragal pumping.

Figure 7 shows the staining level of the middle ear cavity in the TP and control ears. The median (interquartile range) staining levels were 19.52 (10.33, 25.42) in the TP ears and 1.82 (1.04, 6.57) in the control ears defined numbers. Mann‐Whitney *U* test showed that the staining levels were significantly higher in the TP ears than in the control ears (*P* < .001).

### Analysis Methods Validation

**Figure 8:**
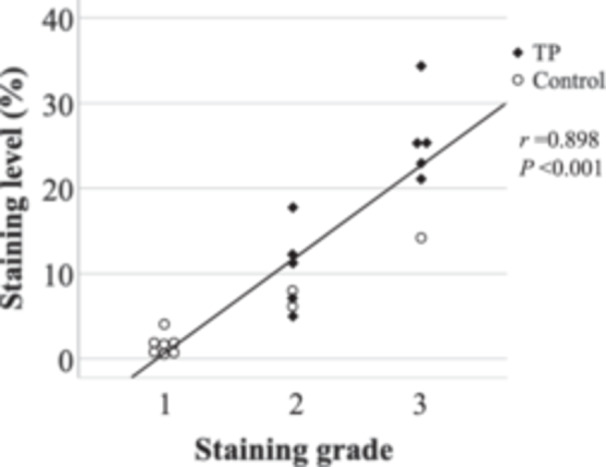
The staining grade and the staining level of the middle ear cavity. Pearson correlation, *r* = .898, *P* < .001. TP, tragal pumping.

Finally, to validate our 2 methods of analysis for the staining of the middle ear cavity, we compared the staining grades of the middle ear cavities and their staining level (Figure 8). There was a significant positive strong correlation between the staining grades of middle ear cavities (histopathology analysis) and their staining level (Image J method) (Pearson correlation, *r* = .898, *P* < .001).

## Discussion

This study investigated the efficacy of the TP in moving fluid from the EAC to the middle ear through the tympanostomy tube in a rat model. Our results showed that a use of TP significantly accelerated the penetration of the dye eardrop solution via a tympanostomy tube that is the same size used in humans.

There are a few previous studies regarding the efficacy of TP in moving solutions into the middle ear cavity by the tympanostomy tube. In an ex vivo human head/ear model, eardrops do not efficiently and consistently pass (up to 4%) through the tympanostomy tube without the use of TP. In contrast, eardrops efficiently move to the middle ear at 100% through the tympanostomy tube with TP.^12^ Yet, in this study, the degree of fluid movement efficacy (how much fluid) was not studied. In a prospective, randomized controlled human study in children with chronic otitis media, the incidence of middle ear cavity penetration of eardrops was significantly higher in the ears with the use of TP (75%) than in control ears (33%).^1^ The results in the previous human studies are similar to the current study as TP can significantly accelerate the penetration of the solutions via a tympanostomy tube. Moreover, if we consider less than 5% of the staining level as an example of a reduced efficiently to move fluid to the middle ear via tympanostomy tubes, in the current study, the incidence of middle ear cavity penetration was 100% in the ears with the use of TP, and 30% in control ears, respectively. In this context, our results showed that performing TP can almost always deliver a certain significant amount of solution into the middle ear. These results of incidence were similar to those in previous studies.^1^, ^12^

The ID of tympanostomy tube is an important factor in our study because solutions can pass more easily through larger tubes than smaller ones.^21^ To investigate the efficacy of TP in the rat model, the ID of tympanostomy tube used in our study was 1.14 mm, the same size that is used in previous human studies.^10^, ^11^ Our application of TP was successful because this tube is the appropriate size to adequately move fluid in contrast to the smaller ID tympanostomy tubes used in rats in the previous studies.^13^, ^14^, ^15^ To insert the tympanostomy tube, the relative stiffness, and type of material of the tube is also important. For the reference in pilot studies, we also tried to insert a fluoroplastic tympanostomy tube (Fluoroplastic Shepard Grommet Ventilation Tube, 1026101, ID: 1.14 mm; Medtronic); however, it could not be placed because of its stiffness. Because this method has never been described before in a rat, we believe this report can also contribute to the establishment of tympanostomy tube‐rat model for further studies. This same‐sized tube should be also useful for other larger species.

The kind of eardrop solution (viscosity) can also affect the results. Previous studies described that the penetration of solutions into the middle ear cavity via the tympanostomy tube is largely governed by surface tension.^2^ We used Davidson® tissue marking dye in this study to detect the penetration of eardrops in the middle ear cavity because other solutions we tried in pilot studies washed out in the tissue processing procedures. Methylene blue was used in a previous human study,^1^ however, it did not work well in our study because the staining disappeared after tissue processing. We also tried hematoxylin solution (VWR International) or crystal violet (Millipore Sigma), however, this dye did not remain intact after tissue processing. Davidson® tissue marking dye is designed to mark surgical biopsy margins and remains intact during tissue processing. The efficacy of TP might also change when different eardrop viscosities are used. Because the procedures for TP ears were performed first, the green dye was in the middle ear cavity of the TP ear longer than that of the control ear. However, we believe it did not affect the results because the green dye used in this study is not for staining the tissue as an immunohistochemistry staining but for marking surgical tissue margins and because we did not evaluate the staining in the tissue but evaluated the green dye in the middle ear cavity. Nonetheless, our results suggest that TP accelerate the penetration of eardrop via tympanostomy tube in the rat.

### Contributions

We believe our results can contribute to the further studies using a tympanostomy tube and eardrop treatments for the middle and inner ear diseases. We have established the novel rat model and the methods to detect the penetration in the middle ear cavity. Because there are few studies describing the efficacy of TP in an animal, we believe our rat model and methods can contribute to the further studies regarding TP with tympanostomy tube, with various eardrops preparations as an alternative method of ITI in patients with middle or inner ear diseases such as ISSNHL^10^ and MD^11^.

## Conclusion

Our findings confirmed that TP significantly accelerated the penetration of the dye solution eardrop into the middle ear cavity in a tympanostomy tube‐rat model. This novel rat model and the methods to detect the penetration in the middle ear cavity can be studied to further the understanding of the efficacy of the eardrop drugs via tympanostomy tube with TP.

## Author Contributions

**Satoshi Hara**, project administration, conceptualization, methodology, formal analysis, investigation, software, data curation, writing—original draft preparation; **Xinyu Liu**, formal analysis; **Firasat Ali Shah**, resources; **Harumi Saeki**, conceptualization; **Hajime Orita**, funding acquisition; **Airi Sakyo**, conceptualization; **Takashi Anzai**, conceptualization; **Yusuke Takata**, conceptualization; **Kazusaku Kamiya**, conceptualization; **Fumihiko Matsumoto**, conceptualization; **Kathleen Gabrielson**, methodology conceptualization, resources, project administration, formal analysis, writing—review and editing, supervision, funding acquisition.

## Disclosures

### Competing interests

The authors have no conflicts of interest to declare.

### Funding source

This work was supported by JSPS KAKENHI Grant Number 24K19757 (to Satoshi Hara). Funding for this research was made possible by Subsidies for Current Expenditures to Private Institutions of Higher Education from the Promotion and Mutual Aid Corporation for Private Schools of Japan, through a subaward from Juntendo University. Its contents are solely the responsibility of the authors and do not necessarily represent the official views of the Promotion and Mutual Aid Corporation for Private Schools of Japan and Juntendo University (as applicable).
